# Poly[tris­(μ_3_-5-amino­isophthalato)diaqua­dicerium(III)]

**DOI:** 10.1107/S160053680803033X

**Published:** 2008-09-27

**Authors:** Hui-Jie Ma, Yu-Hua Fan, Qiang Wang, Cai-Feng Bi, Dong-Mei Zhang

**Affiliations:** a College of Chemistry and Chemical Engineering, Ocean University of China, Shandong 266100, People’s Republic of China

## Abstract

In the title complex, [Ce_2_(C_8_H_5_NO_4_)_3_(H_2_O)_2_]_*n*_, each Ce ion is in nine-coordinated environment. Eight O atoms from six ligands participate in coordination, in addition to one O atom from a water mol­ecule. Both carboxyl­ate groups from the ligands chelate the Ce atoms, forming two four-membered rings. The 5-amino­isophthalate ligands also bridge the Ce centers, forming a two-dimensional network, and O—H⋯O and N—H⋯O hydrogen bonds complete the structure.

## Related literature

For general background, see: Rzaczynska & Belsky (1994 ▶); Daiguebonne *et al.* (2000 ▶); Wu *et al.* (2002*a* ▶,*b* ▶); Liao *et al.* (2004 ▶).
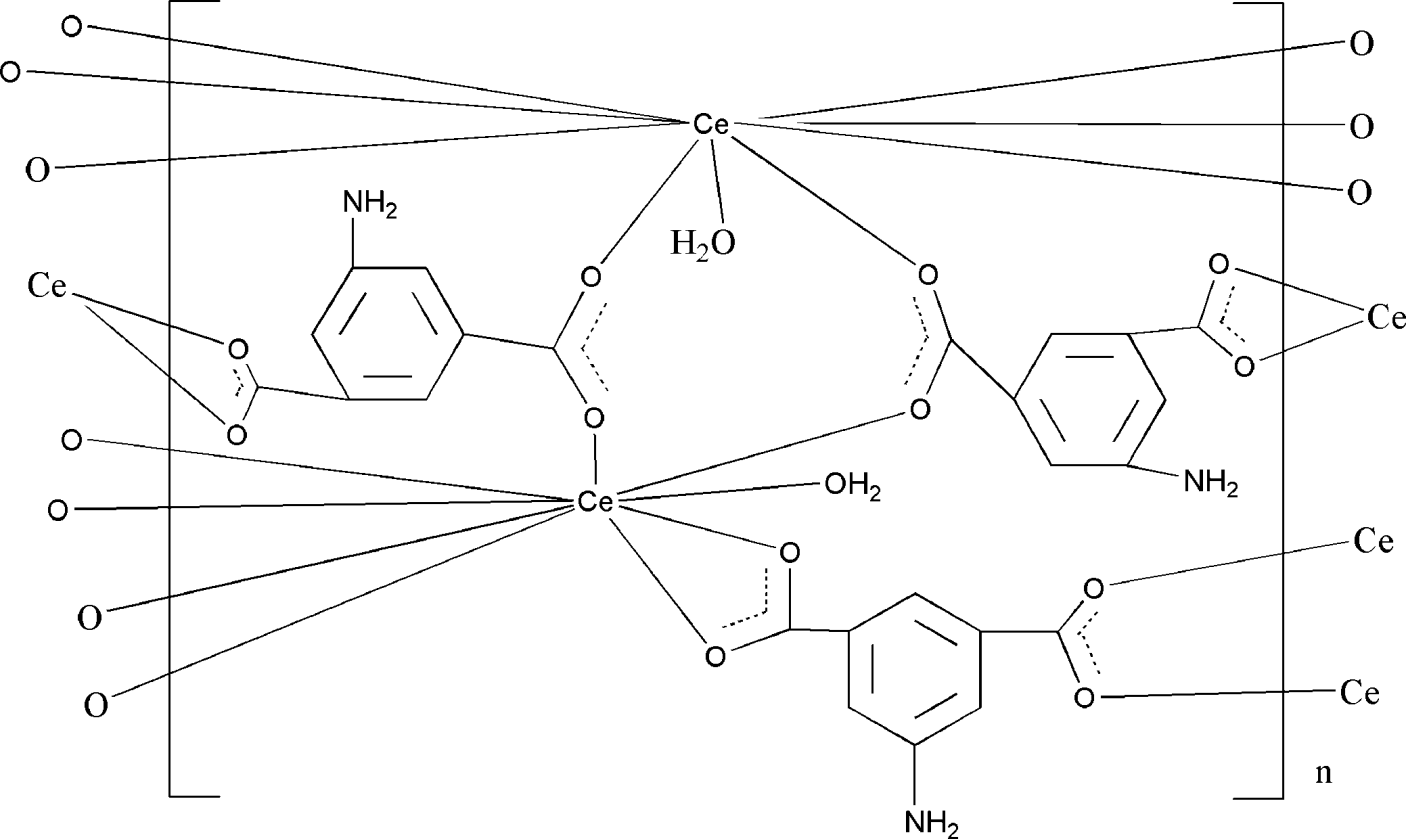


## Experimental

### 

#### Crystal data

[Ce_2_(C_8_H_5_NO_4_)_3_(H_2_O)_2_]
*M* *_r_* = 853.66
Orthorhombic, 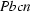

*a* = 12.2360 (7) Å
*b* = 8.0600 (5) Å
*c* = 25.6700 (15) Å
*V* = 2531.6 (3) Å^3^
*Z* = 4
Mo *K*α radiation
μ = 3.63 mm^−1^
*T* = 298 (2) K
0.21 × 0.20 × 0.19 mm

#### Data collection

Siemens SMART CCD area-detector diffractometer
Absorption correction: multi-scan (*SADABS*; Sheldrick, 1996 ▶) *T*_min_ = 0.516, *T*_max_ = 0.545 (expected range = 0.474–0.501)
11818 measured reflections
2233 independent reflections
2001 reflections with *I* > 2σ(*I*)
*R*_int_ = 0.034

#### Refinement

*R*[*F* ^2^ > 2σ(*F* ^2^)] = 0.019
*wR*(*F* ^2^) = 0.045
*S* = 1.04
2233 reflections
196 parameters
2 restraints
H-atom parameters constrained
Δρ_max_ = 0.45 e Å^−3^
Δρ_min_ = −0.64 e Å^−3^

### 

Data collection: *SMART* (Siemens, 1996 ▶); cell refinement: *SAINT* (Siemens, 1996 ▶); data reduction: *SAINT*; program(s) used to solve structure: *SHELXS97* (Sheldrick, 2008 ▶); program(s) used to refine structure: *SHELXL97* (Sheldrick, 2008 ▶); molecular graphics: *SHELXTL* (Sheldrick, 2008 ▶); software used to prepare material for publication: *SHELXTL*.

## Supplementary Material

Crystal structure: contains datablocks I, global. DOI: 10.1107/S160053680803033X/bq2095sup1.cif

Structure factors: contains datablocks I. DOI: 10.1107/S160053680803033X/bq2095Isup2.hkl

Additional supplementary materials:  crystallographic information; 3D view; checkCIF report

## Figures and Tables

**Table 1:** Hydrogen-bond geometry (Å, °)

*D*—H⋯*A*	*D*—H	H⋯*A*	*D*⋯*A*	*D*—H⋯*A*
N2—H2*A*⋯O5^i^	0.86	2.63	3.436 (3)	157
N1—H1*D*⋯O1^ii^	0.86	2.39	3.154 (3)	148
O1*W*—H1*A*⋯O3^i^	0.85	2.07	2.898 (3)	165

Symmetry codes: (i) 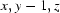; (ii) 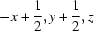.

**Fig. 1.**
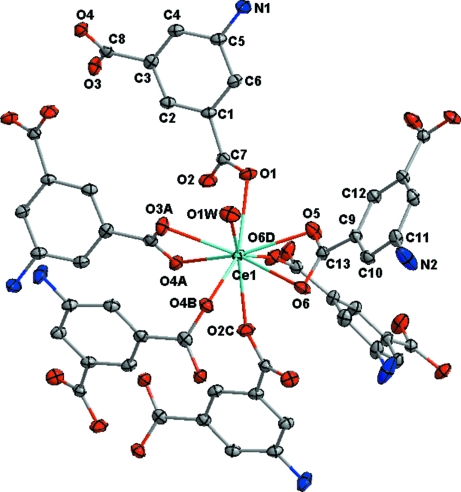
The structure of the title complex, showing 30% probability displacement ellipsoids and the atom-numbering scheme.

## supplementary crystallographic information

### Comment

5-aminoisophthalic acid forms covalent bonds with metal ions, especially with transition metals through nitrogen atom of amino group as well as oxygen atoms of carboxylic groups (Wu *et al.*, 2002a; Wu *et al.*, 2002b; Liao *et al.*, 2004) and with lanthanide ions as strong Pearsons acids through oxygen atoms of carboxylic groups (Rzaczynska *et al.*, 1994). Carboxylic groups of acid have a great ability to form infinite connection with metal ions and remarkable versatility in adopting different modes of bonding-from unidendate, chelating and bridging, sometimes in more than one way in the same compound (Daiguebonne *et al.*, 2000). In this paper, we present a title complex, (C_24_H_19_Ce_2_N_3_O_14_)_n_, (I), synthesized by a condensation reaction of 5-aminoisophthalic acid with cerous nitrate under the condition of high pressure.

The molecular structure of the title complex, (I), is shown in Fig.1. The ligands construct a floor-like layer by chelating and bridging metal ions. The carboxy groups link layers in η^1^,^3^ mode, thus resulting in one-dimension metal-channels along *b*-axis, and the water molecules coordinating with metal ions are pending in these channels.

### Experimental

5-aminoisophthalic acid (0.3 mmol, 54.6 mg) and sodium hydroxide (0.3 mmol,12.5 mg) dissolved in 20 ml water, heated to boiled and then stop heating. Cerous nitrate hexahydrate (0.3 mmol,130.3 mg) dissolved in 5 ml water was mixed with the above solution, stirring for half an hour. Then transfer them into a 50 ml teflon reactor, under autogenous pressure at 160°C for 3 days and then cooled to room temperature, after which large brown block-shaped crystals of the title complex suitable for X-ray diffraction analysis were obtained.

### Refinement

All H-atoms were positioned geometrically and refined using a riding model, with C—H 0.93 (aromatic) 0.93, N—H 0.86 (amino), O—H 0.85 Å (water), with*U*_iso_(H) =1.2*U*_eq_(C).

### Crystal data

**Table:**

[Ce_2_(C_8_H_5_NO_4_)_3_(H_2_O)_2_]	*F*(000) = 1648
*M**_r_* = 853.66	*D*_x_ = 2.240 Mg m^−^^3^
Orthorhombic, *P**b**c**n*	Mo *K*α radiation, λ = 0.71069 Å
Hall symbol: -P 2n 2ab	Cell parameters from 3043 reflections
*a* = 12.2360 (7) Å	θ = 2.3–28.4°
*b* = 8.0600 (5) Å	µ = 3.63 mm^−^^1^
*c* = 25.6700 (15) Å	*T* = 298 K
*V* = 2531.6 (3) Å^3^	Block, brown
*Z* = 4	0.21 × 0.20 × 0.19 mm

### Data collection

**Table:**

Siemens SMART CCD area-detector diffractometer	2233 independent reflections
Radiation source: fine-focus sealed tube	2001 reflections with *I* > 2σ(*I*)
graphite	*R*_int_ = 0.034
φ and ω scans	θ_max_ = 25.0°, θ_min_ = 2.3°
Absorption correction: multi-scan (SADABS; Sheldrick, 1996)	*h* = −14→13
*T*_min_ = 0.516, *T*_max_ = 0.545	*k* = −9→9
11818 measured reflections	*l* = −30→23

### Refinement

**Table:**

Refinement on *F*^2^	Primary atom site location: structure-invariant direct methods
Least-squares matrix: full	Secondary atom site location: difference Fourier map
*R*[*F*^2^ > 2σ(*F*^2^)] = 0.019	Hydrogen site location: inferred from neighbouring sites
*wR*(*F*^2^) = 0.045	H-atom parameters constrained
*S* = 1.04	*w* = 1/[σ^2^(*F*_o_^2^) + (0.0208*P*)^2^ + 2.5416*P*] where *P* = (*F*_o_^2^ + 2*F*_c_^2^)/3
2233 reflections	(Δ/σ)_max_ = 0.001
196 parameters	Δρ_max_ = 0.45 e Å^−^^3^
2 restraints	Δρ_min_ = −0.64 e Å^−^^3^

### Special details

**Table:**

Geometry. All e.s.d.'s (except the e.s.d. in the dihedral angle between two l.s. planes) are estimated using the full covariance matrix. The cell e.s.d.'s are taken into account individually in the estimation of e.s.d.'s in distances, angles and torsion angles; correlations between e.s.d.'s in cell parameters are only used when they are defined by crystal symmetry. An approximate (isotropic) treatment of cell e.s.d.'s is used for estimating e.s.d.'s involving l.s. planes.
Refinement. Refinement of *F*^2^ against ALL reflections. The weighted *R*-factor *wR* and goodness of fit *S* are based on *F*^2^, conventional *R*-factors *R* are based on *F*, with *F* set to zero for negative *F*^2^. The threshold expression of *F*^2^ > σ(*F*^2^) is used only for calculating *R*-factors(gt) *etc*. and is not relevant to the choice of reflections for refinement. *R*-factors based on *F*^2^ are statistically about twice as large as those based on *F*, and *R*- factors based on ALL data will be even larger.

### Fractional atomic coordinates and isotropic or equivalent isotropic displacement parameters (Å^2^)

**Table:**

	*x*	*y*	*z*	*U*_iso_*/*U*_eq_	
Ce1	0.694451 (12)	0.038178 (18)	0.094984 (6)	0.01318 (7)	
H1A	0.4903	−0.1228	0.0487	0.016*	
H1B	0.4671	−0.1140	0.0998	0.016*	
N1	0.1463 (2)	0.4699 (3)	0.15158 (11)	0.0302 (7)	
H1C	0.1531	0.4128	0.1797	0.036*	
H1D	0.1042	0.5534	0.1571	0.036*	
N2	0.5000	−0.7310 (4)	0.2500	0.0418 (11)	
H2A	0.5462	−0.7843	0.2311	0.050*	
O1	0.53914 (15)	0.2297 (2)	0.12641 (8)	0.0227 (5)	
O2	0.62001 (17)	0.4491 (3)	0.09148 (8)	0.0250 (5)	
O3	0.38087 (15)	0.8402 (2)	−0.01169 (8)	0.0220 (5)	
O4	0.77549 (15)	0.2778 (2)	0.03332 (8)	0.0204 (4)	
O5	0.63242 (18)	−0.0423 (2)	0.18383 (9)	0.0282 (5)	
O6	0.70692 (16)	−0.2648 (2)	0.15024 (8)	0.0232 (5)	
O1W	0.51934 (16)	−0.1043 (3)	0.07824 (9)	0.0280 (5)	
C1	0.4286 (2)	0.4564 (3)	0.09890 (11)	0.0159 (6)	
C2	0.4177 (2)	0.5614 (3)	0.05642 (12)	0.0177 (6)	
H2	0.4786	0.5885	0.0365	0.021*	
C3	0.3163 (2)	0.6262 (3)	0.04351 (11)	0.0174 (6)	
C4	0.2262 (2)	0.5922 (4)	0.07468 (12)	0.0179 (6)	
H4	0.1576	0.6316	0.0652	0.021*	
C5	0.2384 (2)	0.4998 (3)	0.11991 (12)	0.0189 (6)	
C6	0.3393 (2)	0.4281 (3)	0.13138 (12)	0.0182 (6)	
H6	0.3469	0.3613	0.1607	0.022*	
C7	0.5365 (2)	0.3728 (3)	0.10682 (11)	0.0164 (6)	
C8	0.3065 (2)	0.7357 (3)	−0.00284 (12)	0.0173 (6)	
C9	0.5727 (2)	−0.2982 (3)	0.21853 (11)	0.0171 (6)	
C10	0.5748 (2)	−0.4714 (3)	0.21935 (12)	0.0205 (6)	
H10	0.6261	−0.5283	0.1995	0.025*	
C11	0.5000	−0.5599 (5)	0.2500	0.0212 (9)	
C12	0.5000	−0.2132 (5)	0.2500	0.0176 (9)	
H12	0.5000	−0.0978	0.2500	0.021*	
C13	0.6425 (2)	−0.1977 (3)	0.18270 (11)	0.0165 (6)	

### Atomic displacement parameters (Å^2^)

**Table:**

	*U*^11^	*U*^22^	*U*^33^	*U*^12^	*U*^13^	*U*^23^
Ce1	0.01069 (10)	0.01242 (10)	0.01642 (12)	−0.00009 (6)	−0.00036 (6)	0.00135 (6)
N1	0.0194 (14)	0.0396 (16)	0.0314 (18)	0.0059 (12)	0.0102 (12)	0.0138 (13)
N2	0.070 (3)	0.0152 (19)	0.040 (3)	0.000	0.030 (2)	0.000
O1	0.0200 (10)	0.0169 (10)	0.0311 (13)	0.0053 (8)	0.0002 (9)	0.0026 (9)
O2	0.0132 (11)	0.0306 (12)	0.0314 (14)	−0.0012 (9)	0.0007 (9)	0.0030 (10)
O3	0.0243 (11)	0.0195 (11)	0.0224 (12)	−0.0062 (9)	−0.0041 (9)	0.0065 (9)
O4	0.0179 (10)	0.0227 (11)	0.0205 (11)	−0.0003 (8)	−0.0036 (9)	0.0016 (9)
O5	0.0395 (13)	0.0170 (11)	0.0280 (13)	0.0005 (9)	0.0133 (11)	0.0036 (9)
O6	0.0235 (11)	0.0225 (9)	0.0236 (12)	0.0025 (9)	0.0101 (9)	0.0048 (8)
O1W	0.0187 (11)	0.0323 (12)	0.0329 (13)	−0.0066 (10)	0.0010 (10)	−0.0076 (11)
C1	0.0136 (14)	0.0137 (14)	0.0204 (16)	−0.0002 (11)	−0.0011 (11)	−0.0010 (12)
C2	0.0151 (14)	0.0154 (14)	0.0226 (16)	−0.0004 (11)	0.0026 (13)	−0.0014 (12)
C3	0.0194 (15)	0.0126 (14)	0.0201 (16)	−0.0010 (11)	−0.0020 (12)	−0.0016 (12)
C4	0.0135 (13)	0.0178 (14)	0.0223 (16)	0.0017 (12)	−0.0013 (12)	−0.0024 (13)
C5	0.0140 (14)	0.0195 (14)	0.0233 (17)	0.0003 (12)	0.0011 (13)	0.0001 (13)
C6	0.0155 (14)	0.0168 (14)	0.0225 (17)	0.0002 (11)	−0.0007 (13)	0.0032 (12)
C7	0.0132 (14)	0.0191 (15)	0.0168 (15)	−0.0005 (12)	−0.0013 (12)	−0.0024 (13)
C8	0.0186 (14)	0.0156 (14)	0.0176 (16)	0.0047 (12)	−0.0009 (12)	−0.0033 (12)
C9	0.0185 (14)	0.0173 (14)	0.0155 (15)	−0.0003 (11)	0.0017 (12)	0.0005 (12)
C10	0.0255 (16)	0.0184 (14)	0.0176 (16)	0.0040 (12)	0.0050 (14)	−0.0020 (13)
C11	0.032 (2)	0.015 (2)	0.016 (2)	0.000	0.0031 (19)	0.000
C12	0.021 (2)	0.016 (2)	0.016 (2)	0.000	0.0012 (17)	0.000
C13	0.0162 (14)	0.0189 (14)	0.0144 (15)	−0.0002 (12)	0.0001 (12)	−0.0004 (12)

### Geometric parameters (Å, °)

**Table:**

Ce1—O2^i^	2.383 (2)	O6—Ce1^i^	2.448 (2)
Ce1—O6^ii^	2.448 (2)	O1W—H1A	0.8500
Ce1—O1W	2.469 (2)	O1W—H1B	0.8500
Ce1—O5	2.490 (2)	C1—C2	1.387 (4)
Ce1—O3^iii^	2.5263 (19)	C1—C6	1.393 (4)
Ce1—O1	2.5779 (19)	C1—C7	1.496 (4)
Ce1—O4^i^	2.654 (2)	C2—C3	1.387 (4)
Ce1—O4	2.6870 (19)	C2—H2	0.9300
Ce1—O6	2.828 (2)	C3—C4	1.389 (4)
Ce1—C8^iii^	2.986 (3)	C3—C8	1.486 (4)
N1—C5	1.410 (4)	C4—C5	1.387 (4)
N1—H1C	0.8600	C4—H4	0.9300
N1—H1D	0.8599	C5—C6	1.395 (4)
N2—C11	1.379 (5)	C6—H6	0.9300
N2—H2A	0.8600	C8—O4^iii^	1.276 (3)
O1—C7	1.258 (3)	C8—Ce1^iii^	2.986 (3)
O2—C7	1.256 (3)	C9—C12	1.384 (3)
O2—Ce1^ii^	2.383 (2)	C9—C10	1.396 (4)
O3—C8	1.261 (3)	C9—C13	1.494 (4)
O3—Ce1^iii^	2.5263 (19)	C10—C11	1.402 (3)
O4—C8^iii^	1.276 (3)	C10—H10	0.9300
O4—Ce1^ii^	2.654 (2)	C11—C10^iv^	1.402 (3)
O5—C13	1.259 (3)	C12—C9^iv^	1.384 (3)
O6—C13	1.268 (3)	C12—H12	0.9300
			
O2^i^—Ce1—O6^ii^	75.39 (7)	C8^iii^—O4—Ce1^ii^	122.81 (17)
O2^i^—Ce1—O1W	132.85 (7)	C8^iii^—O4—Ce1	90.56 (16)
O6^ii^—Ce1—O1W	146.17 (7)	Ce1^ii^—O4—Ce1	105.55 (7)
O2^i^—Ce1—O5	104.28 (7)	C13—O5—Ce1	102.02 (18)
O6^ii^—Ce1—O5	77.79 (7)	C13—O6—Ce1^i^	164.58 (18)
O1W—Ce1—O5	76.92 (7)	C13—O6—Ce1	85.86 (16)
O2^i^—Ce1—O3^iii^	115.63 (7)	Ce1^i^—O6—Ce1	107.23 (7)
O6^ii^—Ce1—O3^iii^	114.74 (6)	Ce1—O1W—H1A	126.9
O1W—Ce1—O3^iii^	73.53 (7)	Ce1—O1W—H1B	125.6
O5—Ce1—O3^iii^	139.93 (7)	H1A—O1W—H1B	104.5
O2^i^—Ce1—O1	153.47 (7)	C2—C1—C6	119.7 (3)
O6^ii^—Ce1—O1	78.08 (6)	C2—C1—C7	117.8 (3)
O1W—Ce1—O1	72.14 (7)	C6—C1—C7	122.5 (2)
O5—Ce1—O1	69.18 (7)	C3—C2—C1	120.2 (3)
O3^iii^—Ce1—O1	76.33 (7)	C3—C2—H2	119.9
O2^i^—Ce1—O4^i^	66.87 (7)	C1—C2—H2	119.9
O6^ii^—Ce1—O4^i^	142.22 (6)	C2—C3—C4	119.9 (3)
O1W—Ce1—O4^i^	69.43 (6)	C2—C3—C8	119.1 (2)
O5—Ce1—O4^i^	112.50 (6)	C4—C3—C8	121.0 (2)
O3^iii^—Ce1—O4^i^	81.52 (6)	C5—C4—C3	120.2 (3)
O1—Ce1—O4^i^	139.66 (6)	C5—C4—H4	119.9
O2^i^—Ce1—O4	80.95 (6)	C3—C4—H4	119.9
O6^ii^—Ce1—O4	72.12 (7)	C4—C5—C6	119.6 (3)
O1W—Ce1—O4	123.51 (7)	C4—C5—N1	119.3 (3)
O5—Ce1—O4	147.09 (6)	C6—C5—N1	121.0 (3)
O3^iii^—Ce1—O4	49.99 (6)	C1—C6—C5	120.0 (3)
O1—Ce1—O4	91.49 (6)	C1—C6—H6	120.0
O4^i^—Ce1—O4	99.54 (6)	C5—C6—H6	120.0
O2^i^—Ce1—O6	72.98 (6)	O2—C7—O1	123.6 (3)
O6^ii^—Ce1—O6	104.06 (7)	O2—C7—C1	117.1 (2)
O1W—Ce1—O6	74.48 (7)	O1—C7—C1	119.3 (2)
O5—Ce1—O6	48.09 (6)	O3—C8—O4^iii^	120.9 (3)
O3^iii^—Ce1—O6	141.19 (6)	O3—C8—C3	118.9 (2)
O1—Ce1—O6	113.57 (6)	O4^iii^—C8—C3	120.2 (2)
O4^i^—Ce1—O6	66.99 (6)	O3—C8—Ce1^iii^	56.79 (15)
O4—Ce1—O6	153.67 (5)	O4^iii^—C8—Ce1^iii^	64.13 (15)
O2^i^—Ce1—C8^iii^	99.07 (7)	C3—C8—Ce1^iii^	175.47 (19)
O6^ii^—Ce1—C8^iii^	93.72 (7)	C12—C9—C10	119.9 (3)
O1W—Ce1—C8^iii^	98.21 (7)	C12—C9—C13	117.3 (3)
O5—Ce1—C8^iii^	152.07 (7)	C10—C9—C13	122.8 (3)
O3^iii^—Ce1—C8^iii^	24.68 (7)	C9—C10—C11	120.4 (3)
O1—Ce1—C8^iii^	83.09 (7)	C9—C10—H10	119.8
O4^i^—Ce1—C8^iii^	90.61 (7)	C11—C10—H10	119.8
O4—Ce1—C8^iii^	25.31 (6)	N2—C11—C10^iv^	120.60 (18)
O6—Ce1—C8^iii^	157.60 (7)	N2—C11—C10	120.60 (18)
C5—N1—H1C	119.9	C10^iv^—C11—C10	118.8 (4)
C5—N1—H1D	116.1	C9^iv^—C12—C9	120.6 (4)
H1C—N1—H1D	109.7	C9^iv^—C12—H12	119.7
C11—N2—H2A	120.0	C9—C12—H12	119.7
C7—O1—Ce1	116.27 (17)	O5—C13—O6	120.0 (3)
C7—O2—Ce1^ii^	155.62 (19)	O5—C13—C9	118.0 (2)
C8—O3—Ce1^iii^	98.53 (17)	O6—C13—C9	121.9 (2)

Symmetry codes: (i) −*x*+3/2, *y*−1/2, *z*; (ii) −*x*+3/2, *y*+1/2, *z*; (iii) −*x*+1, −*y*+1, −*z*; (iv) −*x*+1, *y*, −*z*+1/2.

### Hydrogen-bond geometry (Å, °)

**Table:**

*D*—H···*A*	*D*—H	H···*A*	*D*···*A*	*D*—H···*A*
N2—H2A···O5^v^	0.86	2.63	3.436 (3)	157.
N1—H1D···O1^vi^	0.86	2.39	3.154 (3)	148.
O1W—H1A···O3^v^	0.85	2.07	2.898 (3)	165.

Symmetry codes: (v) *x*, *y*−1, *z*; (vi) −*x*+1/2, *y*+1/2, *z*.
